# Identifying different cognitive phenotypes and their relationship with disability in neuromyelitis optica spectrum disorder

**DOI:** 10.3389/fneur.2022.958441

**Published:** 2022-09-16

**Authors:** Lingyao Kong, Yanlin Lang, Xiaofei Wang, Jiancheng Wang, Hongxi Chen, Ziyan Shi, Hongyu Zhou

**Affiliations:** Department of Neurology, West China Hospital, Sichuan University, Chengdu, China

**Keywords:** neuromyelitis optica spectrum disorder, neuropsychological assessment, cognitive impairment (CI), disability, risk factors, latent profile analysis (LPA)

## Abstract

**Background:**

The existence, frequency, and features of cognitive impairment (CI) in patients with neuromyelitis optica spectrum disorder (NMOSD) are still debated. A precise classification and characterization of cognitive phenotypes in patients with NMOSD are lacking.

**Methods:**

A total of 66 patients with NMOSD and 22 healthy controls (HCs) underwent a neuropsychological assessment. Latent profile analysis (LPA) on cognitive test z scores was used to identify cognitive phenotypes, and ANOVA was used to define the clinical features of each phenotype. Univariate and multivariate analyses were used to explore the predictors of severe CI, and a corresponding nomogram was created to visualize the predictive model.

**Results:**

LPA results suggested four distinct meaningful cognitive phenotypes in NMOSD: preserved cognition (*n* = 20, 30.3%), mild-attention (*n* = 21, 31.8%), mild-multidomain (*n* = 18, 27.3%), and severe-multidomain (*n* = 7, 10.6%). Patients with the last three phenotypes were perceived to have CI, which accounts for 67.6% of patients with NMOSD. Patients with NMOSD and worse cognitive function were older (*p* < 0.001) and had lower educational levels (*p* < 0.001), later clinical onset (*p* = 0.01), worse Expanded Disability Status Scale scores (*p* = 0.001), and poorer lower-limb motor function (Timed 25-Foot Walk, *p* = 0.029; 12-item Multiple Sclerosis Walking Scale [MSWS-12], *p* < 0.001). Deterioration of Nine-Hole Peg Test (odds ratio, OR: 1.115 [1, 1.243], *p* = 0.05) and MSWS-12 (OR: 1.069 [1.003, 1.139], *p* = 0.04) were the independent risk factors for severe cognitive dysfunction. Finally, a nomogram was built based on the entire cohort and the above factors to serve as a useful tool for clinicians to evaluate the risk of severe cognitive dysfunction.

**Conclusions:**

We introduced a classification scheme for CI and highlighted that the deterioration of upper- and lower-limb motor disability potentially predicts cognitive phenotypes in NMOSD.

## Introduction

Neuromyelitis optica spectrum disorder (NMOSD) is an autoimmune inflammatory disease of the central nervous system that has similar symptoms as multiple sclerosis (MS), including recurrent attacks of optic neuritis and myelitis (1). The discovery of aquaporin-4 antibody (AQP4-Ab), a pathogenic autoantibody detectable in the serum of patients with NMOSD, has greatly facilitated the differentiation of the two diseases (2–4).

Both diseases potentially induce progressive cognitive impairment (CI) during their disease courses (5). Previous studies have demonstrated that CI commonly occurs in patients with MS (PwMS) in each cognitive domain and have proposed several classification schemes for cognitive phenotypes (6, 7). However, controversy persists regarding the existence, frequency, and features of CI in patients with NMOSD (8, 9). Variability in the enrolled patient population and cognitive tests potentially influence the results (10). Therefore, we adopted a model-based classification approach using standardized neuropsychological z scores to explore CI features in patients with NMOSD.

In this study, a latent profile analysis (LPA) model, which has been widely used in the fields of neurological and neuropsychiatric disorders over recent years, was applied to elucidate latent cognitive subtypes in NMOSD (6, 11). LPA is a person-centered approach (i.e., assigning patients into subgroups of individuals characterized by similar performances in cognitive tests) (12). In contrast to the variable-based approach, it is a promising approach, as it explores the multiple dimensions of cognitive function in different patterns and how these patterns are related to demographic and clinical characteristics.

This study aimed to (1) identify homogeneous groups of individuals with NMOSD based on the latent profile of their cognitive performance, (2) compare the demographic and clinical characteristics among the identified cognitive phenotypes, and (3) identify independent predictors of severe cognitive dysfunction.

## Materials and methods

### Study design

Between December 2020 and March 2022, 66 patients with NMOSD and 22 healthy controls (HCs) were recruited prospectively from the West China Hospital of Sichuan University. For patients with NMOSD, the inclusion criteria were as follows: (1) diagnosis based on the revised Wingerchuk 2015 criteria (13); (2) age between 16 and 65 years, regardless of sex; (3) no relapses within 30 days; (4) visual acuity ≥20/40 in the better eye; and (5) comprehension of and willingness to participate in this study. Patients excluded from this study were those with (1) severe visual impairment, hearing impairment, or hand-movement disorders, which would affect their performance on cognitive tests; (2) a history of other neuropsychiatric diseases, such as Parkinson's disease, which influence cognitive function; (3) dependence on psychoactive substances, such as alcohol and tobacco; and (4) long-term use of drugs affecting cognitive function, such as benzodiazepines. All patients were AQP4-Ab-positive and myelin oligodendrocyte glycoprotein-antibody-negative, as determined by a commercial cell-based assay (EUROIMMUN AG, Luebeck, Germany) (14–16), and received a long-term immunotherapy at the time of evaluation. HCs were age- and education-matched, with no history of neurological or neuropsychological diseases and substance abuse.

This study was approved by the Medical Ethics Committee of the West China Hospital of Sichuan University (2018 trial no. 29), and written informed consent was obtained from all subjects.

### Neuropsychological assessment

All patients with NMOSD and HCs underwent neuropsychological assessment in a quiet room, and the assessments were performed by an experienced neuropsychologist who was unaware of their clinical diagnoses. Based on the current recommendations for the evaluation of cognitive function in MS (17), we used seven cognitive tests to measure three main cognitive domains in this study. Each domain was assessed using at least two to three cognitive tests to ensure the comprehensiveness of the evaluation. Visuospatial and verbal memory were assessed using the Brief Visuospatial Memory Test-Revised (BVMT-R) (18) and California Verbal Learning Test-Second Edition (CVLT-II) (19), respectively. The Digit Span Forward and Backward (DST-F&DST-B) (20), Symbol Digit Modalities (SDMT) (21), and Paced Auditory Serial Addition (PASAT, interstimulus time, 3 s) tests (22) were used to assess attention and the speed of information processing. In addition, the executive clock drawing task (CLOX) (23) and Wisconsin card sorting test (WCST) (24) were administered to evaluate executive function. Fatigue and depression were evaluated using the Chinese version of the Brief Fatigue Inventory (BFI) (25) and Patient Health Questionnaire-9 (PHQ-9) (26), respectively. The details of the abovementioned tests and scales have been described in our previous studies (27).

For further analysis, raw scores of each cognitive test were transformed into z scores, which were calculated using the following formula: (patient's score - mean value of the matched HCs/standard deviation (SD) of the matched HCs). For each cognitive test, CI was defined as 1 SD below the mean value (28). In this study, CI was described as “mild” (z scores between−1 and−1.5 SD), “moderate” (z scores between−1.5 and−2 SD), and “severe” (z scores less than −2 SD) (29).

### Clinical evaluation

For enrolled patients with NMOSD, we collected the following information and entered it into our database: age, sex, education years, disease duration, history of relapses, annual relapse rate (ARR), number of severe attacks, medications, and presence of lesions on brain and/or spinal cord magnetic resonance imaging (MRI). Expanded Disability Status Scale (EDSS) scores were evaluated by two independent and blinded neurological physicians on the same day as the neuropsychological assessment. The Nine-Hole Peg Test of the dominant and nondominant hand (NHPT-D & NHPT-N), Timed 25-Foot Walk (T25FW), and the 12-item Multiple Sclerosis Walking Scale (MSWS-12) were applied to evaluate upper- and lower-extremity function quantitatively.

A severe attack was defined as an EDSS score of ≥6 at the nadir of the attack or an increase of ≥0.5 points if the patient had a baseline EDSS score of ≥6. For patients with optic neuritis, a severe relapse was defined as a new worsening of visual acuity of ≤ 0.1 at the nadir of the attack. If baseline vision was light perception, hand motion, or counting fingers, any decrease with MRI evidence of optic neuritis was considered a severe relapse (30).

### Statistical analysis

To identify the cognitive phenotypes in patients with NMOSD, we performed LPA of cognitive test z-scores. LPA is a type of Gaussian mixture modeling that can identify latent classes in a dataset of continuous variables. A series of models with different numbers of “latent” classes are estimated and compared in LPA, and the optimally fitting model is selected based on the following model-fit indices (Supplementary Table 1): (1) the Akaike information criterion and Bayesian information criterion, in which a smaller value represents a better fit; (2) the bootstrap likelihood ratio test, in which *p* < 0.05 indicates that the K_n_-class model provides a significantly better fit than the K_n−1_-class model; and (3) entropy, whereby a higher value represents a better fit, with values of >0.80 indicating highly discriminating latent classes.

The demographic and clinical characteristics at baseline were analyzed using analysis of variance or the Kruskal–Wallis test for continuous variables and the Chi-squared test for categorical variables. Fisher's Least Significant Difference (homogeneous variances) or Games Howell (heterogeneous variances) test was used for *post hoc* comparison. The above statistical analyses were performed using SPSS (version 23.0; IBM, Armonk, NY, USA) and plotted in GraphPad Prism (version 7.0, GraphPad Prism Software, La Jolla, CA, USA 7.0). Statistical significance was set at *P* < 0.05. LPA was performed with Mplus (version 8.0, Los Angeles, CA, USA) and R software (version 4.1.2, R Foundation for Statistical Computing, Vienna, Austria) using the “tidyLPA,” “tidyverse,” “mclust,” and “MplusAutomation” packages. Univariate analysis of the generalized ordered logistic regression model was used to screen for CI-relevant significant variables. Factors with *p* < 0.05 in the univariate analysis, as well as several clinically important factors, were used in the multivariate analysis. A nomogram was constructed using the regression coefficients (β) from the ordinal logistic regression model using R packages “rms”.

## Results

### Cognitive phenotypes

Table 1 shows the baseline characteristics of patients who are AQP4-Ab-positive with NMOSD and HCs. No significant differences were found regarding age, gender, education level, depression, and fatigue scores.

**Table 1:** Baseline characteristics of patients with NMOSD and healthy controls.

	**AQP4^+^ NMOSD (*n =* 66)**	**HC** **(*n =* 22)**	***p*-value**
Age, Mean (SD)	37.4 (12.2)	37.1 (8.9)	0.895
Female, n (%)	54 (81.8)	17 (65.4)	0.104
Education, Mean (SD)	12.3 (4.3)	12.9 (3.8)	0.561
ARR, Median (IQR)	0.9 (0.5, 1.3)	-	-
Disease duration, Median (IQR)	3.4 (1.4, 9.9)	-	-
EDSS score, Mean (SD)	2.5 (1.8)	-	-
PHQ-9, Mean (SD)	5.8 (4.0)	6.1 (4.7)	0.778
BFI, Mean (SD)	19.2 (17.2)	26.9 (17.7)	0.127
Immunotherapy, n (%)			
Low-dose of Prednisone (Oral)	5 (7.6)	-	-
Mycophenolate mofetil	43 (65.2)	-	-
Rituximab	14 (21.2)	-	-
Azathioprine	4 (6.1)	-	-

**Figure 1:**
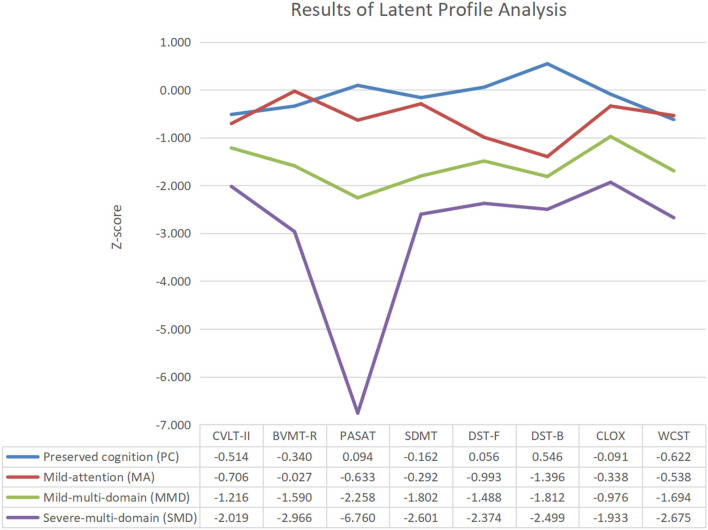
Results of the latent profile analysis.

LPA identified four homogeneous cognitive phenotypes in patients with NMOSD (Figure 1): (1) preserved cognition (PC, *n* = 20, 30.3%), which refers to those who performed well in each cognitive test with the difference from HCs being within the acceptable tolerance (<1 SD below the mean HC score); (2) mild-attention (MA, *n* = 21, 31.8%), which refers to those with mild impairment in DST-B compared with HCs; (3) mild-multidomain (MMD, *n* = 18, 27.3%), which refers to those who were mildly impaired in CVLT-II, BVMT-R, SDMT, DST-F, DST-B, CLOX, and WCST, as well as moderately impaired in PASAT compared with HCs; and (4) severe-multidomain (SMD, *n* = 7, 10.6%)., which refers to those who were mildly impaired in CLOX, moderately impaired in CVLT-II, BVMT-R, SDMT, DST-F, DST-B, and WCST; and severely impaired in PASAT. The four phenotypes were ranked in order of CI severity, and patients with the last three phenotypes were perceived to have CI.

Compared with the last two groups, patients with PC and MA had preserved executive function and could be distinguished by their performance with regard to attention. Among attention test scores in the first two groups, a more pronounced decrease was observed in DST-B (z scores: from 0.509 to −1.349), followed by DST-F (z scores: from 0.025 to −0.867), PASAT (z scores: from −0.021 to −0.620), and SDMT (z scores: from −0.189 to −0.360). Patients with MMD and SMD exhibited various severities of CI in each domain and were characterized by impaired executive function.

### Clinical features of different cognitive phenotypes

Statistical differences were predominantly observed in patients with MMD/SMD phenotypes compared with those in patients with PC/MA phenotypes (Table 2, Supplementary Figure 1).

**Table 2:** The demographic and clinical characteristics of the four cognitive phenotypes.

**Variables**	**Preserved cognition**	**Mild-attention**	**Mild-multi-domain**	**Severe-multi-domain**	***p*-values**
	**(*n =* 20)**	**(*n =* 21)**	**(*n =* 18)**	**(*n =* 7)**	
Age, Mean (SD)	31.6 (9.0)^**a**^	34.3 (11.3)^**b**^	41.6 (12.3)^**a, b**^	52.1 (7.7)^**a, b**^	0.001*
Female, n (%)	15 (75.0)	18 (85.7)	15 (83.3)	6 (85.7)	0.882
Education years, Median (IQR)	16.0 (12.5, 16.0)^**a, b**^	12.0 (9.0, 15.8)	10.5 (5.8, 14.6)^**a**^	9.0 (9.0, 10.0)^**b**^	<0.001*
Disease duration, Median (IQR)	2.4 (1.3, 4.9)	3.3 (1.2, 9.4)	4.4 (1.7, 11.1)	13.0 (3.5, 18.2)	0.066
ARR, Median (IQR)	1.0 (0.6, 1.6)	0.9 (0.7, 1.5)	0.6 (0.4, 0.9)	0.7(0.4, 1.3)	0.216
EDSS, Median (IQR)	1.5 (0.3, 3.0)^**a**^	2.0 (1.5, 3.0)^**b**^	3.0 (1.9, 4.0)	4.0 (3.5, 5.5)^**a, b**^	0.001*
NHPT, Median (IQR)					
Dominant Hand	20.1 (18.2, 24.2)	20.5 (18.6, 23.0)	21.0 (20.4, 24.0)	24.4 (20.3, 36.7)	0.222
Non-dominant Hand	22.5 (20.9, 24.0)	20.4 (19.2, 22.7)	22.7 (19.9, 27.2)	27.2 (30.0, 41.4)	0.101
T25FW, Median (IQR)	5.3 (4.8, 5.9)	5.2 (4.6, 6.3)^**a**^	5.2 (4.9, 7.0)	7.0 (6,4, 8.8)^**a**^	0.045*
MSWS-12, Median (IQR)	1.0 (0, 15.1)^**a, b**^	0 (0, 11.5)^**c, d**^	16.7 (5.7, 40.1)^**a, c**^	43.8 (18.8, 66.7)^**b, d**^	<0.001*
PHQ-9, Median (IQR)	7.0 (5.0, 9.0)	4.5 (2.8, 7.5)	5.0 (3.0. 7.8)	6.3 (4.9)	0.691
BFI, Median (IQR)	13.0 (9.0, 25.0)	12.5 (7.3, 23.3)	15.0 (7.5, 28.8)	29.0 (0, 50.0)	0.765
Age at onset, Mean (SD)	27.0 (9.1)^**a, b**^	29.0 (12.3)^**c**^	35.6 (13.1)^**a**^	40.9 (11.5)^**b, c**^	0.018*
EDSS at onset, Median (IQR)	3.0 (2.0, 5.5)	3.0 (2.0, 5.3)	4.0 (3.0, 7.0)	4.0 (3.0, 8.5)	0.181
Number of attacks, Median (IQR)	2.5 (2.0, 3.0)	3.0 (2.0, 3.5)	3.0 (1.0, 5.0)	5.0 (3.0, 9.0)	0.135
Number of severe attacks, Median (IQR)	0 (0, 1.0)	1.0 (0, 2.0)	0.5 (0, 1.0)	0 (0, 1.0)	0.394
Immunotherapy, n (%)					0.264
Low-dose of Prednisone (Oral)	2 (10.0)	1 (4.8)	2 (11.1)	0	
Mycophenolate mofetil	15 (75.0)	13 (61.9)	12 (66.7)	3 (42.9)	
Rituximab	2 (10.0)	7 (33.3)	3 (16.7)	2 (28.6)	
Azathioprine	1 (5.0)	0	1 (5.6)	2 (28.6)	
Number of patients with MRI lesions, n (%)					
Brain	9 (52.9)	9 (60.0)	12 (85.7)	4 (80.0)	0.228
Cervical cord	11 (68.8)	7 (46.7)	14 (87.5)	5 (83.3)	0.076
Thoracic cord	6 (46.2)^**a**^	5 (41.7)^**b**^	11 (91.7)^**a, b**^	4 (80.0)	0.035*

*Represents p <0.05.

p-values are the result of ANOVA (normal distribution) and Kruskal–Wallis ANOVA (non-normal distribution).

For *post hoc* testing, Fisher's Least Significant Difference (homogeneous variances) or Games Howell (heterogeneous variances) test was used. Significant differences (p <0.05) between any two groups is indicated with the same superscript letters.

The results of *post hoc* testing are shown in Supplementary Figure 1.

The bold *p* values indicate statistical significance.

Regarding demographic characteristics, patients with MMD/SMD were older and had lower educational levels than those with PC (MMD vs. PC: mean age, *p* = 0.005, median education, *p* = 0.003; SMD vs. PC: mean age, *p* < 0.001, median education, *p* = 0.007) and MA (mean age: MMD vs. MA:0.038; SMD vs. MA: *p* < 0.001). No significant differences in depression (PHQ-9, *p* = 0.691) and fatigue (BFI, *p* = 0.765) were noted among the four phenotypes.

In terms of clinical features, patients with MMD/SMD had an older age of onset than those with PC (MMD vs. PC, *p* = 0.025; SMD vs. PC, *p* = 0.008] and MA (MMD *vs*. MA, *p* = 0.081; SMD *vs*. MA, *p* = 0.022). A higher proportion of patients with thoracic cord involvement was noted in patients with worse cognitive functions (*p* = 0.035). The EDSS score at onset, ARR, disease duration, and number of severe attacks were similar among the four phenotypes. There was no difference in CI severity in patients with different immunotherapies.

Regarding disability assessment, significant differences were observed between patients with MMD/SMD and patients with PC/MA, where higher EDSS (SMD vs. PC, *p* = 0.001; SMD vs. MA, *p* = 0.008), T25FW (SMD vs. MA, *p* = 0.039), and MSWS-12 (MMD vs. PC, *p* = 0.015; SMD vs. PC, *p* = 0.006; MMD vs. MA, *p* = 0.013; SMD vs. MA, *p* = 0.005) scores indicated poorer lower-limb function. No significant difference in the upper limb function (NHPT-D, *p* = 0.222; NHPT-N, *p* = 0.101) was observed; thus precluding the possible interferences of writing speed in cognitive tests, especially in SDMT.

### Disability correlates with cognitive phenotypes

**Figure 2:**
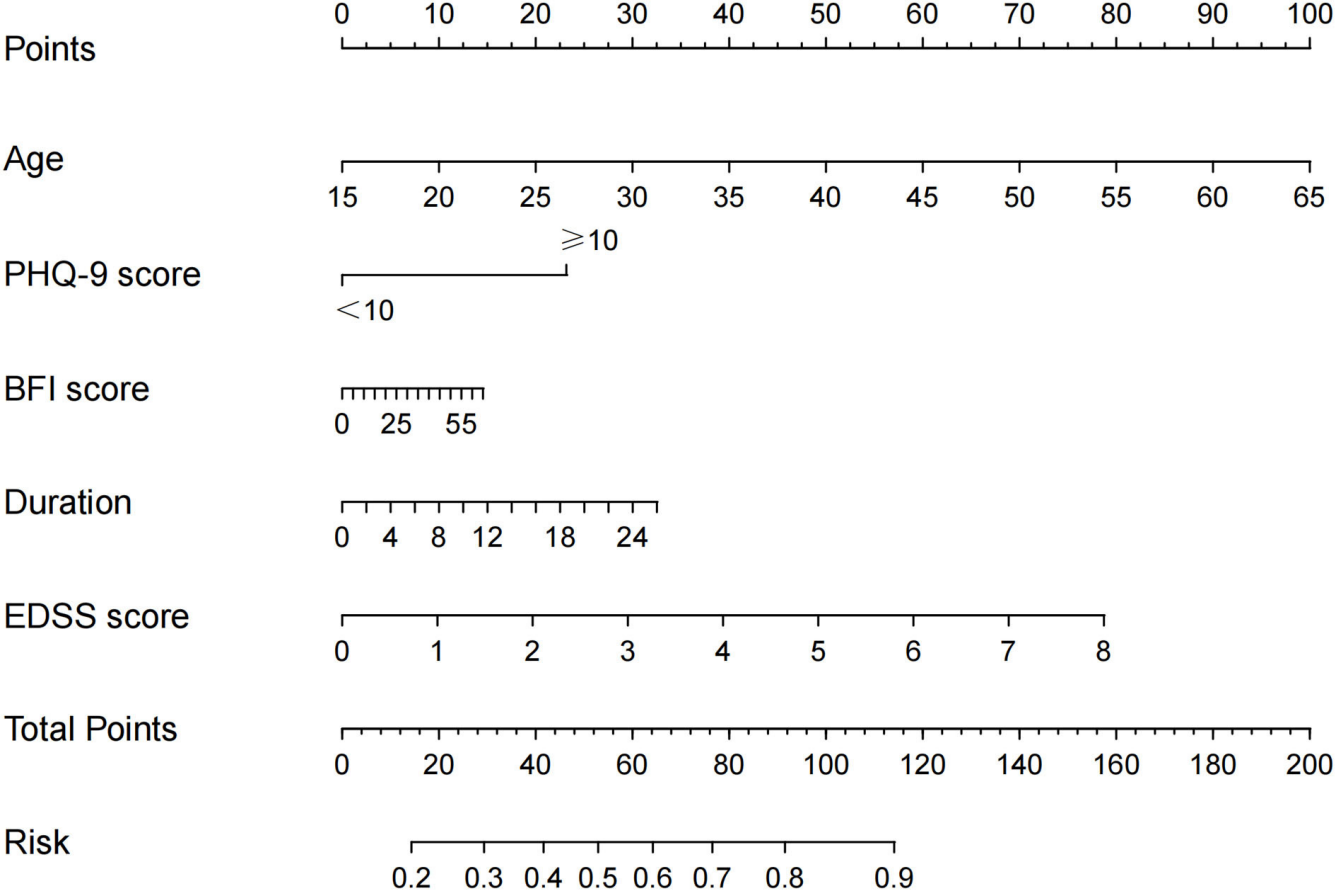
A nomogram for predicting cognitive phenotypes in patients with NMOSD.

Univariate analysis revealed six factors that were associated with cognitive phenotypes, and four of them (EDSS, NHPT, T25FW, and MSWS-12) were used to quantify disability (Table 3). Association between disability and cognitive phenotypes were investigated separately, adjusting for age, education, immunotherapy, disease duration, PHQ-9, and BFI (Table 3, Supplementary Table 2). Multivariate analysis demonstrated that older age (OR: 1.071 [1.013, 1.132], *p* = 0.015), deterioration in EDSS (OR: 1.758 [1.172, 2.636], *p* = 0.006), and MSWS-12 (OR: 1.112 [0.709, 1.744], *p* = 0.004) scores had independent negative impacts on CI severity in patients with NMOSD. A nomogram for this model was formulated (Figure 2), which is a graphical calculation tool that can provide individual risk scores for each patient.

**Table 3:** Independent predictors for severe CI according to univariate and multivariate analysis of generalized ordered logistic regression model.

	**Univariate analyses**			**Multivariate analyses**		
**Variables**	**OR**	**95% CI**	***p* value**	**OR**	**95% CI**	***p* value**
Age	1.087	(1.043, 1.133)	<0.001*	1.071	(1.013, 1.132)	0.015*
Education						
Educational level = 1	7.213	(0.618, 84.205)	0.115	4.153	(0.249, 69.412)	0.322
Educational level = 2	3.117	(0.261, 37.175)	0.369	5.296	(0.352, 79.734)	0.228
Educational level = 3	0.932	(0.088, 9.869)	0.953	0.597	(0.040, 8.932)	0.708
Educational level = 4	0.689	(0.076, 6.215)	0.740	0.551	(0.047, 6.397)	0.633
Educational level = 5	1	-	-	1	-	-
Male	0.641	(0.200, 2.056)	0.454	-	-	-
PHQ-9	1.013	(0.900, 1.141)	0.826	1.057	(0.885, 1.262)	0.542
BFI	1.010	(0.991, 1.048)	0.190	0.993	(0.950, 1.038)	0.750
Disease duration	1.086	(1.000, 1.179)	0.050	1.048	(0.962, 1.141)	0.285
ARR	0.514	(0.256, 1.033)	0.062	-	-	-
Immunotherapy						
Low-dose of Prednisone	0.555	(0.088, 3.503)	0.530	0.602	(0.068, 5.339)	0.649
Mycophenolate mofetil/ Azathioprine	0.692	(0.244, 1.969)	0.490	0.172	(0.033, 0.890)	0.036*
Rituximab	1	-	-	1	-	-
EDSS	1.758	(1.314, 2.354)	<0.001*	1.758	(1.172, 2.636)	0.006*
NHPT						
Dominant Hand	1.119	(1.006, 1.244)	0.038*	-	-	-
Non-dominant Hand	1.130	(1.013, 1.261)	0.028*	-	-	-
T25FW	1.423	(1.068, 1.897)	0.016*	-	-	-
MSWS-12	1.044	(1.021, 1.067)	<0.001*	-	-	-
Age at onset	1.058	(1.019, 1.098)	0.003*	-	-	-
EDSS at onset	1.161	(0.985, 1.369)	0.075	-	-	-
Number of attacks	1.156	(0.979, 1.364)	0.087	-	-	-
Number of severe attacks	1.290	(0.913, 1.822)	0.149	-	-	-

(Education was categorized into five levels: “1” = primary schooling, “2” = secondary schooling, “3” = high school, “4” =college education, and “5” = graduate education).

*Represents p <0.05.

The bold *p* values indicate statistical significance.

## Discussion

A recently published systematic review demonstrated that the pooled prevalence of CI was estimated as 44% in NMOSD, which had a wide range from 3 to 75% among the different studies (31). Although it seems that NMOSD involves structural changes in gray matter and white matter networks, it remains difficult to link CI with one specific tissue alteration (10). Several previous studies have reported decreased cognitive performance in memory, attention, and executive function (27, 32, 33); thus, highlighting the need for cognitive assessment in patients with NMOSD. In this study, we identified latent cognitive phenotypes using LPA, clarified the detailed characteristics of the different cognitive phenotypes, and explored the predictors of severe cognitive dysfunction in patients with NMOSD. To increase the homogeneity of patients, we only enrolled patients with AQP4-Ab positive and MOG-Ab negative results.

Our LPA results suggest the possibility of four distinct, meaningful cognitive phenotypes in NMOSD: “PC,” “MA,” “MMD,” and “SMD” (Figure 1). The denomination of the abovementioned four cognitive phenotypes was referenced from a recent study on multiple sclerosis, which reported five cognitive phenotypes in PwMS using the same LPA method (6). Previous studies have indicated that the frequency and patterns of CI differ between NMOSD and MS (5), and the main finding of our study further supports this view. Our results revealed that CI occurs in 67.6% of patients with NMOSD, especially regarding the evaluation of attention, working memory, and information processing speed, in line with previous evidence (34). However, De Meo et al. reported a higher CI percentage of 80.6% in PwMS, which was characterized by a greater deficiency in verbal learning and memory operation. This discrepancy may be due to a difference in structural brain damage between the two diseases. Less-severe atrophy in the deep gray matter potentially contributes to the relatively superior maintenance of memory function in patients with NMOSD to that in PwMS (35, 36).

We found that patients with NMOSD and worse cognitive function were older (*p* = 0.001) and had lower educational levels (*p* < 0.001), later clinical onset (*p* = 0.018), worse EDSS scores (*p* = 0.001), and poorer lower-limb motor function (T25FW, *p* = 0.045; MSWS-12, *p* < 0.001). Further univariate and multivariate analyses confirmed that age, EDSS scores, and MSWS-12 deteriorations were independent risk factors for severe CI. The MSWS-12 measures different aspects of walking, such as running and climbing stairs, and has been suggested to be more responsive to changes than either the EDSS scores or T25FW (37, 38). The NHPT has been demonstrated to be potentially applicable to patients with NMOSD, exhibiting strong correlations with EDSS scores (39). Einarsson et al. concluded that lower disability was predictive of the capacity to perform the NHPT and walk 10 m in PwMS (40). Chronic pain, a frequent and one of the most disabling symptoms of NMOSD, was found to negatively affect the quality of life in a recent study (41). A further study on the association between pain and cognitive function is warranted (41).

In this study, older age predicted an increased risk of severe CI. To eliminate other confounding factors, we excluded patients with age-related comorbidities that may affect cognitive function, such as cardiovascular and cerebrovascular diseases, dementia, and vascular risk factors, such as diabetes mellitus, hypertension, and hyperlipidemia. The detailed information about comorbidities is summarized in Supplementary Table 3. The proportion of patients with comorbidities did not differ among the four cognitive phenotypes, which might have minimized the potential confounding effects of comorbidities to a certain degree. Although our results revealed that age at onset was not a predictor of severe CI, a pediatric study of 67 children with NMOSD found that a younger age at onset was associated with CI (42). Camera et al. also reported that age at onset could predict first relapse and long-term disabilities (43). No differences in self-reported depression and fatigue were noted among the different cognitive phenotypes, a finding that is consistent with those of previous studies (44). Furthermore, we analyzed certain clinical characteristics that have never been reported before, such as EDSS score at onset, number of previous attacks, and severe attacks. Among them, the number of previous attacks was identified as a risk factor for recurrent relapses (45). Despite the lack of statistical differences in our data, our findings indicate that CI is probably not an accumulation of previous episodes but a reflection of the current disability status.

This study has certain limitations. First, the names of different cognitive phenotypes were decided by the researchers according to the patients' performance in cognitive tests; thus, potentially bearing a degree of subjectivity. Current knowledge does not allow for a complete understanding of the meaning of these phenotypes. Second, no recognized battery of cognitive tests was available to measure cognition in patients with NMOSD, and such variability might have affected the study outcomes. Third, our results may not be generalized to patients with NMOSD and concomitant severe impairments of vision and upper-limb function, which constitute major clinical symptoms in NMOSD and affect a certain proportion of patients. Finally, our results have not been verified in an independent external cohort. Due to the limited sample size and cross-sectional design, we recommend caution in drawing practical clinical conclusions. Large-scale studies involving MRI analysis are required to verify our conclusion and provide neuroanatomical support for the classification of cognitive phenotypes.

## Data availability statement

The raw data supporting the conclusions of this article will be made available by the authors, without undue reservation.

## Ethics statement

The study was approved by Institutional Review Board of West China Hospital, Sichuan University (2018 Trial No. 29). The patients/participants provided their written informed consent to participate in this study.

## Author contributions

LK: methodology, investigation, formal analysis, data curation, visualization, and writing-original draft. YL, JW, HC, and ZS: methodology, investigation, and data curation. XW: methodology, investigation, and formal analysis. HZ: conceptualization, validation, investigation, writing-review and editing, and supervision. All authors contributed to the article and approved the submitted version.

## Funding

This work was funded by the Natural Science Foundation of Sichuan Province (Grant No. 2022NSFSC1432 to XW), Department of Science and Technology of Sichuan Province (Grant Nos. 2020YFS0219 to HC and 2021YFS0173 to ZS), and 1·3·5 project for disciplines of excellence–Clinical Research Incubation Project, West China Hospital, Sichuan University (Grant No. 21HXFH041 to HZ).

## Conflict of interest

The authors declare that the research was conducted in the absence of any commercial or financial relationships that could be construed as a potential conflict of interest.

## Publisher's note

All claims expressed in this article are solely those of the authors and do not necessarily represent those of their affiliated organizations, or those of the publisher, the editors and the reviewers. Any product that may be evaluated in this article, or claim that may be made by its manufacturer, is not guaranteed or endorsed by the publisher.
