# Supplementary material for: Identifying different cognitive phenotypes and their relationship with disability in neuromyelitis optica spectrum disorder
Source: Front Neurol. 2022 Sep 16;13:958441. doi: 10.3389/fneur.2022.958441 (PMC9524354; doi:10.3389/fneur.2022.958441)
Supplement: Supplementary file 1 [file Table_1.DOCX]

| Number of profiles | 1 | 2 | 3 | 4 | 5 |
| --- | --- | --- | --- | --- | --- |
| AIC | 1698.61 | 1541.82 | 1515.22 | 1488.75 | 1493.37 |
| BIC | 1730.49 | 1591.90 | 1583.52 | 1575.27 | 1598.10 |
| Entropy | 1.00 | 0.92 | 0.90 | 0.89 | 0.91 |
| BLRT | - | 0.01 | 0.01 | 0.01 | 0.34 |

S. Table 1. Model fit indices for the LPA.

AIC: Akaike information criterion; BIC: Bayesian information criterion; BLRT: bootstrap likelihood ratio test

S. Table 2. Multivariate analysis of generalized ordered logistic regression model.

|  | **NHPT** | | **T25FW** | | **MSWS-12** | |
| --- | --- | --- | --- | --- | --- | --- |
| **Variables** | **OR 95% CI** | ***p* value** | **OR 95% CI** | ***p* value** | **OR 95% CI** | ***p* value** |
| Age | 1.104  (1.041, 1.171) | **0.001*** | 1.101  (1.041, 1.165) | **0.001*** | 1.072  (1.014, 1.133) | **0.015*** |
| Education |  |  |  |  |  |  |
| Educational level=1 | 1.448  (0.095, 22.137) | 0.790 | 2.452  (0.150, 40.071) | 0.529 | 1.813  (0.112, 29.323) | 0.675 |
| Educational level=2 | 2.392  (0.173, 33.061) | 0.515 | 3.436  (0.219, 53.852) | 0.379 | 2.304  (0.156, 34.117) | 0.544 |
| Educational level=3 | 0.367  (0.021, 6.283) | 0.489 | 0.657  (0.039, 10.946) | 0.770 | 0.373  (0.025, 5.495) | 0.472 |
| Educational level=4 | 0.423  (0.038, 4.732) | 0.485 | 0.647  (0.053, 7.981) | 0.734 | 0.278  (0.024, 3.219) | 0.306 |
| Educational level=5 | 1 | - | 1 | - | 1 | - |
| PHQ-9 | 1.041  (0.850, 1.276) | 0.698 | 1.099  (0.911, 1.325) | 0.325 | 1.047  (0.879, 1.248) | 0.606 |
| BFI | 1.020  (0.977, 1.064) | 0.376 | 1.003  (0.959, 1.049) | 0.889 | 0.994  (0.951, 1.038) | 0.776 |
| Disease duration | 1.051  (0.962, 1.149) | 0.272 | 1.082  (0.978, 1.197) | 0.128 | 1.097  (0.968, 1.150) | 0.221 |
| Immunotherapy |  |  |  |  |  |  |
| Low-dose of Prednisone | 1.194  (0.106, 13.460) | 0.886 | 0.812  (0.076, 8.691) | 0.863 | 0.911  (0.106, 7.810) | 0.932 |
| Mycophenolate mofetil/ Azathioprine | 0.380  (0.067, 2.142) | 0.273 | 0.225  (0.042, 1.216) | 0.083 | 0.224  (0.045, 1.131) | 0.070 |
| Rituximab | 1 | - | 1 | - | 1 | - |
| NHPT | 1.060  (0.947, 1.185) | 0.311 |  |  |  |  |
| T25FW |  |  | 1.269  (0.860, 1.871) | 0.230 |  |  |
| MSWS-12 |  |  |  |  | 1.112  (0.709, 1.744) | **0.004*** |

(Education was categorized into five levels: “1” =primary schooling, “2” =secondary schooling, “3” =high school, “4” =college education, and “5” = graduate education.)

*Represents p < 0. 05.

S. Table 3. The comorbidities of patients among four cognitive phenotypes.

| **Variables** | **Preserved cognition**  **(n=20)** | **Mild-attention**  **(n=21)** | **Mild-multi-domain**  **(n=18)** | **Severe-multi-domain**  **(n=7)** | ***p*-values** |
| --- | --- | --- | --- | --- | --- |
| Comorbidity | 7 (35.0) | 8 (38.1) | 10 (55.6) | 3 (42.9) | 0.617 |
| Respiratory diseases | 1 | 1 | 0 | 0 |  |
| Digestive diseases | 1 | 2 | 3 | 0 |  |
| Endocrine diseases | 4 | 3 | 0 | 2 |  |
| Hematologic diseases. | 0 | 1 | 1 | 0 |  |
| Other autoimmune diseases | 1 | 5 | 6 | 1 |  |

Respiratory Diseases: one patient has suffered tuberculosis and been cured, and another is chronic rhinitis.

Digestive diseases: gastroesophageal reflux disease and non-alcoholic fatty liver disease.

Endocrine Diseases: hypothyroidism and Hashimoto’s thyroiditis.

Hematologic diseases: iron deficiency anemia.

Other autoimmune diseases: including Sjögren’s syndrome, Hashimoto’s thyroiditis, Raynaud’s disease, and the idiopathic thrombocytopenic purpura.

S. Figure 1. The demographic and clinical characteristics of the four cognitive phenotypes.


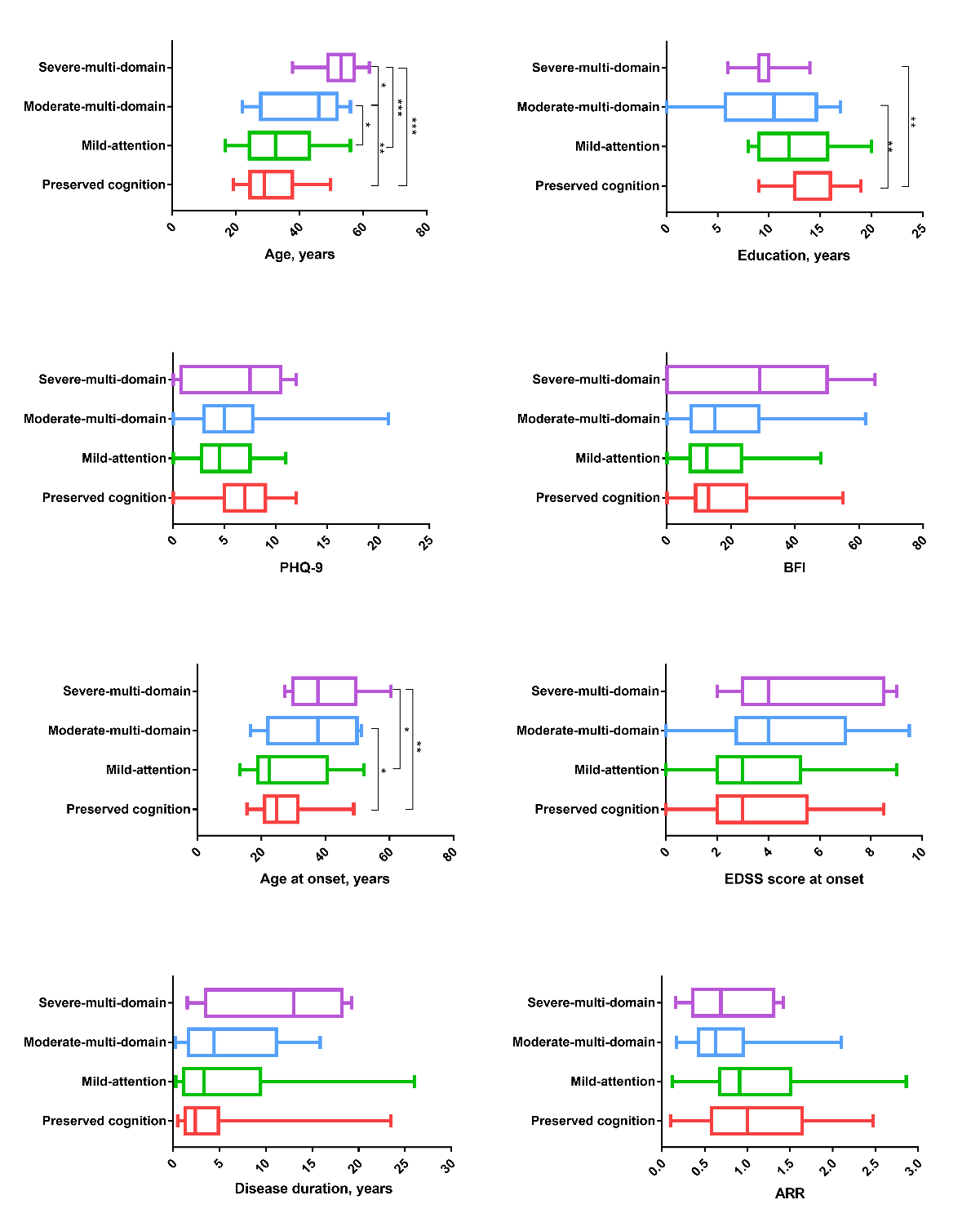


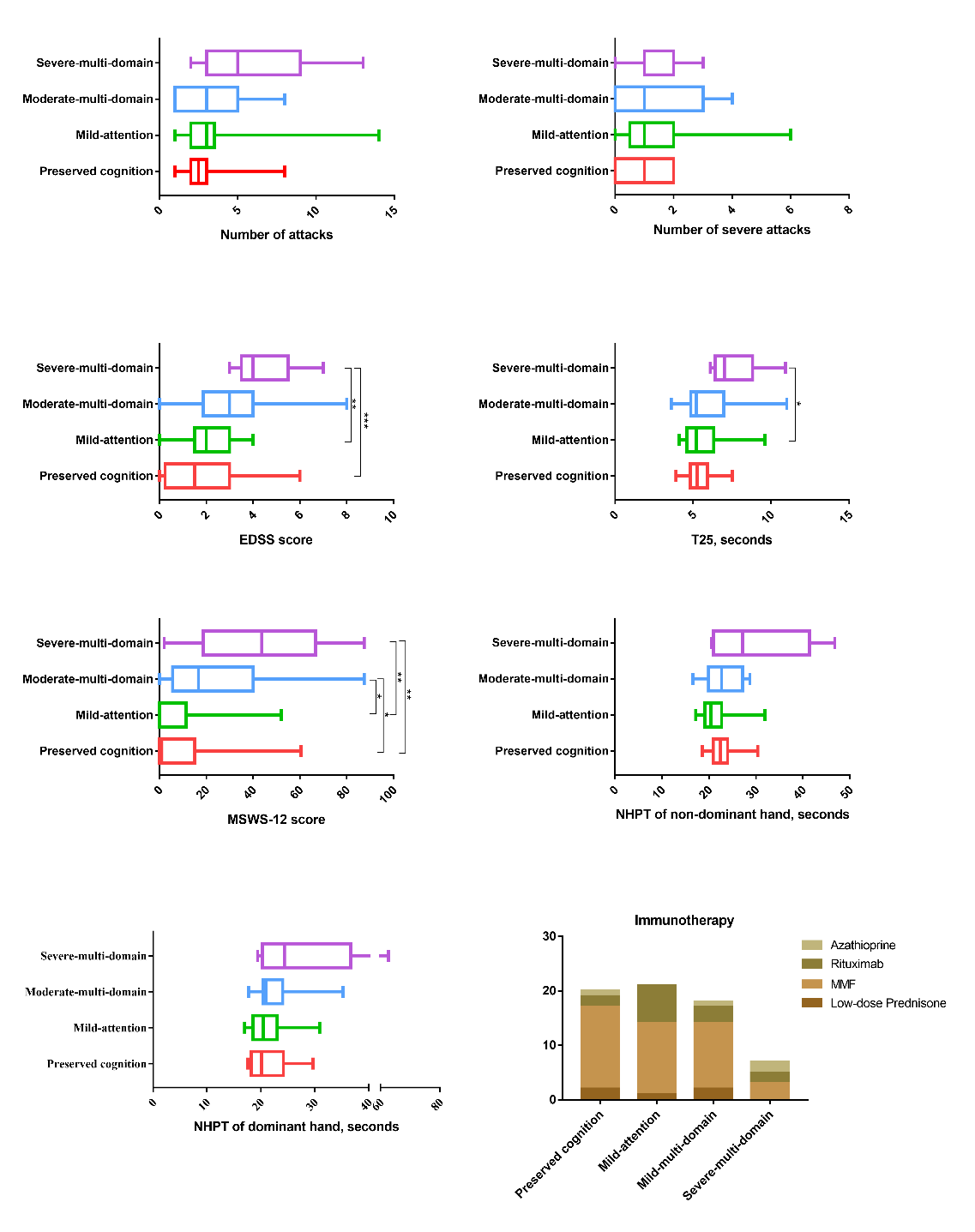


Significant differences (p < 0. 05) between any two groups in post hoc testing are indicated with asterisks. * Represents p < 0.05, ** represents p < 0.01 and *** represents p < 0.001.
